# Supplementary material for: Fluoroquinolone Metalloantibiotics: A Promising Approach against Methicillin-Resistant Staphylococcus aureus
Source: Int J Environ Res Public Health. 2020 Apr 30;17(9):3127. doi: 10.3390/ijerph17093127 (PMC7246690; doi:10.3390/ijerph17093127)
Supplement: Supplementary file 1 [file ijerph-17-03127-s001.pdf]

Table S1. Antibiotic resistance profiles of the MDR isolates of *E. coli* and *P. aeruginosa* used in microbiological assays, determined according to EUCAST guidelines [1].

Characteristics	Bacterial Strain					
	**Ec1-SA1** [a]	**Ec2-SA1** [a]	**Ec3-SA1** [a]	**Ec4-SA1** [a]	**HSJ Ec001**	**HSJ Ec002**
**Sample**	unknown	unknown	unknown	unknown	unknown	unknown
**Resistant to**	ATM-CAZ-CTX-TE-AMP-SXT-CPX-GEN	ATM-CAZ-CTX-TE-AMP-CPX	CAZ-CTX-TE-AMP-CPX-GEN	ATM-CTX-AMP	AMP- CXM-CF-CPX-SXT-LVX	AMC-AMP-CAZ-CXM-CTX-CPX-GEN-TZP-TOB-SXT-LVX
	**Bacterial Strain**					
**Characteristics**	**HSJ Ec003**	**HSJ Ec004**	**Pa1-SA2** [b]	**Pa2-SA2** [b]	**Pa3-SA2** [b]	**Pa4-SA2** [b]
**Sample**	unknown	unknown	unknown	unknown	unknown	unknown
**Resistant to**	CXM-CPX-LVX	AMC-AMP-CXM-CPX-TZP-LVX	CAZ-IPM-CPX	IPM-GEN-CPX-FEP	GEN-CPX-FEP	CAZ-IPM-CPX-FEP

[a] Strains were tested against AMC-ATM-CAZ-CTX-TE-CHL-AMP-FOX-SXT-CPX-IPM-GEN. [b] Strains were tested against: CAZ-ATM-IPM-GEN-CPX-FEP. **Antibiotic abbreviation:** AMC - Amoxicillin-clavulanate; AMP – Ampicillin; ATM – Aztreonam; CAZ – Ceftazidime; CF - Cephalothin; CHL – Chloramphenicol; CPX - Ciprofloxacin; CTX - Cefotaxime; CXM - Cefuroxime; DA - Clindamycin; DAP – Daptomycin; E - Erythromycin; FEP – Cefepime; FOX – Cefoxitin; GEN - Gentamicin; IPM – Imipenem; LNZ – Linezolid; LVX - Levofloxacin; OX - Oxacillin; RD – Rifampin; SXT – Trimethoprim-sulfamethoxazole; TE – Tetracycline; TEC – Teicoplanin; TGC – Tigecycline; TOB – Tobramycin; TZP – Piperacillin-tazobactam; VA – Vancomycin.

Table S2. Antibiotic resistance profiles of the MRSA isolates used in microbiological assays, determined according to EUCAST guidelines [1].

Characteristics	Bacterial Strain					
	**Sa1-SA3** [c]	**Sa2-SA3** [c]	**Sa3-SA3** [c]	**Sa4-SA3** [c]	**17/05** [d]	**17/08** [d]
**Sample**	-	-	-	-	P.E.G. (cateter)	foreskin buffer
**Resistant to**	VA-AMP-FOX-CPX-OX	AMC-AMP-FOX-IPM-CPX-OX	VA-AMC-AMP-FOX-IPM-CPX-OX	AMP-FOX-CPX-OX	OXA-CTX-DA-E-CPX-LEV	OXA-CTX-GEN-DA-E-CPX-LEV-DAP^{NS}
**Clone**	-	-	-	-	ST228/SCCmecIV	ST228/SCCmecI
**Characteristics**	**Bacterial Strain**					
	**37/3** [d]	**38/13 bis** [d]	**59/57** [d]	**27/17** [d]	**5/41** [d]	**58/01** [d]
**Sample**	bronchial lavage	umbical cord	buffer wound	unknown	emoculture	buffer wound
**Resistant to**	OXA-CTX-GEN-E-CPX-LEV	OXA-CTX-GEN-CPX-LEV	OXA-CXT-DA-E-CPX-LEV-DAP^{NS}-LNZ	CTX-CPX-LEV	OXA-CXT-GEN-DA-E-CPX-LEV-DAP^{NS}-LNZ	OXA-CXT-CPX-LEV-RD
**Clone**	ST22/SCCmecIV.h	ST22/SCCmecIV.h	ST22/SCCmecIV.h	IV.h/ST22/E-MRSA15/t20	ST5/SCCmecII	ST5/SCCmecII

[c]All strains were tested against: SXT-VA-AMC-CHL-TE-AMP-FOX-IPM-GEN-CPX-OX.
[d] All strains were tested against: OXA-CTX-GEN-DA-E-CPX-LEV-LNZ-DAP-TGC-RD-VA-TEC.

Table S2. Continued.

Characteristics	Bacterial Strain					
	**26/01 [d]**	**16/01 [d]**	**6/16 bis [d]**	**3/146 [d]**	**7/21 bis [d]**	**19/35 [d]**
**Sample**	unknown	scalp injury	buffer wound	ulcer	emoculture	pus
**Resistant to**	OXA-CTX-GEN-CPX-LEV	OXA-CTX-GEN-CPX-LEV	OXA-CXT-DA-E-CPX-LEV-DAP^{NS}-RD	OXA-CTX-GEN-E-CPX-LEV	OXA-CXT-GEN-CPX-LEV	OXA-CXT-GEN-DA-E-CPX-LEV-DAP^{NS}-RD
**Clone**	ST239/SCCmecIII	ST772/SCCmec IV.c PVL+	ST5/SCCmecII	ST8/SCCmecIV	ST8/SCCmecIV	ST63/SCCmecIV

[d] All strains were tested against: OXA-CTX-GEN-DA-E-CPX-LEV-LNZ-DAP-TGC-RD-VA-TEC. **Antibiotic abbreviation:** AMC - Amoxicillin-clavulanate; AMP – Ampicillin; ATM – Aztreonam; CAZ – Ceftazidime; CF - Cephalothin; CHL – Chloramphenicol; CPX - Ciprofloxacin; CTX - Cefotaxime; CXM - Cefuroxime; DA - Clindamycin; DAP – Daptomycin; E - Erythromycin; FEP – Cefepime; FOX – Cefoxitin; GEN - Gentamicin; IPM – Imipenem; LNZ – Linezolid; LVX - Levofloxacin; OX - Oxacillin; RD – Rifampin; SXT - Trimethoprim-sulfamethoxazole; TE – Tetracycline; TEC – Teicoplanin; TGC – Tigecycline; TOB – Tobramycin; TZP – Piperacillin - tazobactam; VA – Vancomycin.

Table S3. Molecular weight of cpx, erx, lvx, mxfx, spx and respective CuFQphen complexes, expressed in g mol^{-1}. The molecular weights of metalloantibiotics were calculated based on crystallographic data previously available [2].

Antibiotic	Molecular weight (g mol^{-1})	
	**FQ**	**CuFQphen**
cpx	331.34	709.16 [a]
erx	359.39	674.05 [b]
lvx	361.37	721.18 [c]
mxfx	437.89	788.26 [d]
spx	392.4	770.22 [e]

[a] $[Cu(cpx)(phen)](NO_3).4H_2O$; [b] $[Cu(erx)(phen)]Cl_2$; [c] $[Cu(lvx)(phen)(H_2O)]NO_3.2H_2O$; [d] $[Cu(mxfx)(phen)]NO_3.4.5H_2O$; [e] $[Cu(spx)(phen)H_2O]NO_3.3H_2O$.

Table S4. MIC values of several FQs, CuFQphen complexes, phen, Cu(II)/phen (1:1) and $Cu(NO_3)_2.3H_2O$ salt against reference strains, *E. coli* ATCC 25922, *P. aeruginosa* ATCC 27853, *S. aureus* ATCC 25923 and *S. aureus* ATCC 29213, expressed in μg mL^{-1} and μmol dm^{-3} for comparative purposes. The values presented were obtained from at least three independent experiments.

Compound	MIC value							
	***E. coli* ATCC 25922**		***P. aeruginosa* ATCC 27853**		***S. aureus* ATCC 25923**		***S. aureus* ATCC 29213**	
	**μg mL^{-1}**	**μmol dm^{-3}**	**μg mL^{-1}**	**μmol dm^{-3}**	**μg mL^{-1}**	**μmol dm^{-3}**	**μg mL^{-1}**	**μmol dm^{-3}**
cpx	0.004	0.012	0.06	0.18	0.12–0.25	0.36–0.75	0.25–0.5	0.75–1.51
erx	0.008	0.022	1	2.78	0.12–0.25	0.33–0.70	0.12	0.33
lvx	0.008	0.022	0.5	1.38	0.12–0.25	0.33–0.69	0.12	0.33
mxfx	0.008	0.018	0.5 - 1	1.14–2.28	0.03–0.06	0.07–0.14	0.06	0.14
spx	0.004	0.010	0.25–0.5	0.64–1.27	0.03–0.06	0.08–0.15	0.06	0.15
Cucpxphen	0.008	0.011	0.12–0.25	0.17–0.35	0.25–0.5	0.35–0.71	1	1.41
Cuerxphen	0.015	0.022	2	2.97	0.25–0.5	0.37–0.74	0.12–0.25	0.18–0.37
Culvxphen	0.015–0.03	0.021–0.042	1	1.39	0.25–0.5	0.35–0.69	0.25	0.35
Cumxfxphen	0.015–0.03	0.019–0.038	2	2.54	0.06–0.12	0.08–0.15	0.06–0.12	0.08–0.15
Cuspxphen	0.004–0.008	0.005–0.010	0.5	0.65	0.25	0.32	0.06–0.12	0.08–0.16
phen	8	40.4	128	645.7	16 - 32	80.7–161.4	32	161.4
Cu(II)/phen (1:1)	32	72.8	≥512	≥1164.1	32	72.8	64	145.5
$Cu(NO_3)_2.3H_2O$	≥1024	≥4238.2	≥1024	≥4238.2	≥1024	≥4238.2	≥880	≥3642.2

Table S5. MIC values of several FQs, CuFQphen complexes, phen, Cu(II)/phen (1:1) and $Cu(NO_3)_2.3H_2O$ salt against eight MDRR isolates of *E. coli* (Ec1-SA1, Ec2-SA1, Ec3-SA1, Ec4-SA1, HSJ Ec001, HSJ Ec002, HSJ Ec003 and HSJ Ec004), expressed in μg mL^{-1} and μmol dm^{-3} for comparative purposes. The values presented were obtained from at least three independent experiments.

Compound	MIC value

	Ec1-SA1		Ec2-SA1		Ec3-SA1		Ec4-SA1	
	**μg mL^{-1}**	**μmol dm^{-3}**	**μg mL^{-1}**	**μmol dm^{-3}**	**μg mL^{-1}**	**μmol dm^{-3}**	**μg mL^{-1}**	**μmol dm^{-3}**
cpx	1	3.0	64	193.2	4	12.1	16	48.3
erx	0.5–1	1.4–2.8	32	89.0	8	22.3	32–64	89.0–178.1
lvx	0.5	1.4	8–16	22.1–44.3	4	11.1	8	22.1
mxfx	0.5–1	1.1–2.3	8	18.3	4	9.1	8–16	18.3–36.5
spx	4	10.2	32–64	81.6–163.1	8	20.4	32	81.6
Cucpxphen	2	2.8	64	90.3	8	11.3	64	90.3
Cuerxphen	2–4	3.0–5.9	64	95.0	16	23.7	64	95.0
Culvxphen	1–2	1.4–2.8	64–128	88.7–177.5	8	11.1	16–32	22.2–44.4
Cumxfxphen	1–2	1.3–2.5	32–64	40.6–81.2	8	10.2	16–32	20.3–40.6
Cuspxphen	4	5.2	64–128	83.1–166.2	16	20.8	64	83.1
phen	8–16	40.4–80.7	8	40.4	8	40.4	8	40.4
Cu(II)/phen (1:1)	32–64	72.8–145.5	32	72.8	32–64	72.8–145.5	32	72.8
$Cu(NO_3)_2.3H_2O$	>1024	>4238.2	≥1024	≥4238.2	≥1024	≥4238.2	≥1024	≥4238.2

Table S5. Continued.

**Compound**	**MIC value**							
	**HSJ Ec001**		**HSJ Ec002**		**HSJ Ec003**		**HSJ Ec004**	
	**μg mL^{-1}**	**μmol dm^{-3}**	**μg mL^{-1}**	**μmol dm^{-3}**	**μg mL^{-1}**	**μmol dm^{-3}**	**μg mL^{-1}**	**μmol dm^{-3}**
cpx	16	48.3	128	386.3	64	193.2	8	24.1
erx	16	44.5	64	178.1	64	178.1	8	22.3
lvx	8	22.1	8	22.1	32	88.6	4	11.1

mxfx	4	9.1	8	18.3	8	18.3	2	4.6
spx	8	20.4	16	40.8	32	81.6	4	10.2
Cucpxphen	64	90.3	64	93.0	32–64	45.1–90.3	16	22.6
Cuerxphen	64	95.0	64	95.0	64	95.0	16	23.7
Culvxphen	16	22.2	32	44.4	32–64	44.4–88.7	8	11.1
Cumxfxphen	8	10.2	16	20.3	32	40.6	4	5.1
Cuspxphen	32	41.6	32	41.6	64	83.1	8	10.4
phen	8	40.4	16	80.7	8	40.4	8	40.4
Cu(II)/phen (1:1)	32	72.8	64	145.5	32	72.8	32	72.8
$Cu(NO_3)_2.3H_2O$	>1024	>4238.2	>1024	>4238.2	≥1024	≥4238.2	≥1024	≥4238.2

Table S6. MIC values of several FQs, CuFQphen complexes, phen, Cu(II)/phen (1:1) and $Cu(NO_3)_2.3H_2O$ salt against four MDR isolates of *P. aeruginosa* (Pa1-SA2, Pa2-SA2, Pa3-SA2 and Pa4-SA2), expressed in μg mL^{-1} and μmol dm^{-3} for comparative purposes. The values presented were obtained from at least three independent experiments.

Compound	MIC value							
	Pa1-SA2		Pa2-SA2		Pa3-SA2		Pa4-SA2	
	μg mL^{-1}	μmol dm^{-3}	μg mL^{-1}	μmol dm^{-3}	μg mL^{-1}	μmol dm^{-3}	μg mL^{-1}	μmol dm^{-3}
cpx	0.5	1.5	2–4	6.0–12.1	8	24.1	8–16	24.1–48.3
erx	4	11.1	8	22.3	32	89.0	32–64	89.0–178.1
lvx	2	5.5	4	11.1	8	22.1	32	88.6
mxfx	4	9.1	8	18.3	16	36.5	16	36.5
spx	4–8	10.2–20.4	8	20.4	32	81.6	16–32	40.8–81.6
Cucpxphen	1–2	1.4–2.8	4–8	5.6–11.3	16–32	22.6–45.1	16–32	22.6–45.1
Cuerxphen	8	11.9	32	47.5	128–256	189.9–379.8	256	379.8

Culvxphen	4	5.6	8	11.1	32	44.4	32	44.4
Cumxfxphen	8	10.2	32	40.6	64	81.2	32	40.6
Cuspxphen	8	10.4	16–32	20.8–41.6	128	166.2	16–32	20.8–41.6
phen	16	80.7	64	322.9	64	322.9	32–64	161.4–322.9
Cu(II)/phen (1:1)	64–128	145.5–291.0	256	582.0	>512	>1164.1	256	582.0
$Cu(NO_3)_2.3H_2O$	1024	4238.2	1024	4238.2	1024	4238.2	1024	4238.2

Table S7. MIC values of several FQs, CuFQphen complexes, phen, Cu(II)/phen (1:1) and $Cu(NO_3)_2.3H_2O$ salt against 18 MRSA isolates (Sa1-SA3, Sa2-SA3, Sa3-SA3, Sa4-SA3, 17/05, 17/08, 37/3, 38/13 bis, 59/57, 27/17, 5/41, 58/01, 6/16 bis, 3/146, 7/21 bis, 19/35, 26/01 and 16/01), expressed in μg mL^{-1} and μmol dm^{-3} for comparative purposes. The values presented were obtained from at least three independent experiments.

Compound	MIC value							
	Sa1-SA3		Sa2-SA3		Sa3-SA3		Sa4-SA3	
	μg mL^{-1}	μmol dm^{-3}	μg mL^{-1}	μmol dm^{-3}	μg mL^{-1}	μmol dm^{-3}	μg mL^{-1}	μmol dm^{-3}
cpx	128	386.3	128	386.3	128–256	386.3–772.6	8	24.1
erx	8	22.3	128	356.2	16 - 32	44.5–89.0	4	11.1
lvx	256	708.4	64	177.1	16	44.3	4	11.1
mxfx	8	18.3	16	36.5	128	292.3	2	4.6
spx	256	652.4	128	326.2	8	20.4	4	10.2
Cucpxphen	64	90.3	32–64	45.1–90.3	64	90.3	16	22.6
Cuerxphen	64	95.0	32–64	47.5–95.0	64	95.0	8	11.9
Culvxphen	64	88.7	64	88.7	64–128	88.7–177.5	8	11.1
Cumxfxphen	8	10.2	16	20.3	8	10.2	8	10.2
Cuspxphen	64–128	83.1–166.2	32–64	41.6–83.1	32	41.6	16	20.8

phen	512	2582.9	32	161.4	128	645.7	128	645.7
Cu(II)/phen (1:1)	32	72.8	32	72.8	32	72.8	32	72.8
$Cu(NO_3)_2.3H_2O$	≥1024	≥4238.2	1024	4238.2	≥1024	≥4238.2	≥1024	≥4238.2

Table S7. Continued.

**Compound**	**MIC value**							
	**17/05**		**17/08**		**37/3**		**38/13 bis**	
	**μg mL^{-1}**	**μmol dm^{-3}**	**μg mL^{-1}**	**μmol dm^{-3}**	**μg mL^{-1}**	**μmol dm^{-3}**	**μg mL^{-1}**	**μmol dm^{-3}**
cpx	≥256	772.6	32–64	96.6–193.2	≥512	≥1545.2	128–512	386.3–1545.2
erx	4–8	11.1–22.3	8–16	22.3–44.5	32	89.0	8	22.3
lvx	16	44.3	8–16	22.1–44.3	64	177.1	8–16	22.1
mxfx	4	9.1	2	4.6	8	18.3	2	4.6
spx	16	40.8	32	81.6	16–32	40.8–81.6	16–32	40.8
Cucpxphen	128	180.5	64–128	90.3–180.5	128	180.5	128	180.5
Cuerxphen	8–16	11.9–23.7	16	23.7	64	95.0	16	23.7
Culvxphen	8–32	11.1–44.4	16	22.2	64	88.7	32	44.4
Cumxfxphen	4–8	5.1–10.2	4	5.1	16	20.3	4–8	5.1–10.2
Cuspxphen	32	41.6	16	20.8	16	20.8	16–32	20.8–41.6
phen	256–512	1291.4–2582.9	32	16.4	64–128	322.9–645.7	64	322.9
Cu(II)/phen (1:1)	64	145.5	64–128	145.5–291.0	64	145.5	64	145.5
$Cu(NO_3)_2.3H_2O$	≥880	≥3642.2	≥880	≥3642.2	≥880	≥3642.2	≥880	≥3642.2

Table S7. Continued.

Compound	MIC value							
	59/57		27/17		5/41		58/01	
	μg mL^{-1}	μmol dm^{-3}	μg mL^{-1}	μmol dm^{-3}	μg mL^{-1}	μmol dm^{-3}	μg mL^{-1}	μmol dm^{-3}
cpx	≥1024	≥3090.5	≥1024	≥3090.5	64	193.2	32	96.6
erx	32	89.0	64	178.1	64–128	178.1–356.2	8–16	22.3–44.5
lvx	32	88.6	64–128	177.1–354.2	32	88.6	8–16	22.1–44.3
mxfx	4	9.1	4–8	9.1–18.3	8	18.3	8	18.3
spx	32	81.6	16–32	40.8–81.6	64	163.1	32	81.6
Cucpxphen	128	180.5	128	180.5	128	180.5	64	90.3
Cuerxphen	64	95.0	64	95.0	64	95.0	16	23.7
Culvxphen	64	88.7	64	88.7	64	88.7	16	22.2
Cumxfxphen	8	10.2	16	20.3	16	20.3	4	5.1
Cuspxphen	32	41.6	16–32	20.8–41.6	32	41.6	16	20.8
phen	128	645.7	64	322.9	128	645.7	256	1291.4
Cu(II)/phen (1:1)	32–64	72.8–145.5	64	145.5	64	145.5	64	145.5
$Cu(NO_3)_2.3H_2O$	≥880	≥3642.2	≥880	≥3642.2	≥880	≥3642.2	≥880	≥3642.2

Table S7. Continued.

Compound	MIC value							
	6/16 bis		3/146		7/21 bis		19/35	
	μg mL^{-1}	μmol dm^{-3}	μg mL^{-1}	μmol dm^{-3}	μg mL^{-1}	μmol dm^{-3}	μg mL^{-1}	μmol dm^{-3}
cpx	32–64	96.6–193.2	64–128	193.2–386.3	256	772.6	≥1024	≥3090.5

erx	16	44.5	8	22.3	8	22.3	64	178.1
lvx	16	44.3	16–32	44.3–88.6	32	88.6	64	177.1
mxfx	2	4.6	4	9.1	4	9.1	8	18.3
spx	32	81.6	16–32	40.8–81.6	32	81.6	64	163.1
Cucpxphen	64	90.3	64–128	90.3–180.5	128	180.5	128	180.5
Cuerxphen	16	23.7	16	23.7	16	23.7	64	95.0
Culvxphen	16	22.2	32	44.4	32	44.4	64	88.7
Cumxfxphen	4	5.1	8	10.2	8	10.2	16	20.3
Cuspxphen	16	20.8	16	20.8	16	20.8	32	41.6
phen	128	645.7	32	161.4	32	161.4	64	322.9
Cu(II)/phen (1:1)	64	145.5	32–64	72.8–145.5	64	145.5	64	145.5
$Cu(NO_3)_2.3H_2O$	≥880	≥3642.2	≥880	≥3642.2	≥880	≥3642.2	≥880	≥3642.2

Table 7. Continued.

**Compound**	**MIC value**			
	**26/01**		**16/01**	
	**μg mL^{-1}**	**μmol dm^{-3}**	**μg mL^{-1}**	**μmol dm^{-3}**
cpx	512	1545.2	16	48.3
erx	256	712.3	2–8	5.6–22.3
lvx	512	1416.8	4–8	11.1–22.1
mxfx	32	73.1	2	4.6
spx	256	652.4	4–8	10.2–20.4
Cucpxphen	128	180.5	64	90.3

Cuerxphen	64	95.0	8	11.9
Culvxphen	64	88.7	8–16	11.1–22.2
Cumxfxphen	32	40.6	4	5.1
Cuspxphen	64	83.1	8–16	10.4–20.8
phen	64–128	322.9–645.7	32	161.4
Cu(II)/phen (1:1)	64	145.5	64	145.5
$Cu(NO_3)_2.3H_2O$	≥880	≥3642.2	≥880	≥3642.2

Table S8. Growth inhibition zones caused by Cucpxphen, Cuspxphen, phen, Cu(II)/phen (1:1) and $Cu(NO_3)_2.3H_2O$ salt against a MDR isolate of *E. coli* (HSJ Ec002). The diameter of the zones of growth inhibition is presented in mm. The values presented were obtained from at least two independent experiments.

**Compound**	**Compound alone**	**Compound + cpx**	**Compound + amp**
	**MDR *E. coli* HSJ Ec002**		
Cucpxphen	0	0	0
Cuspxphen	0	8	0
phen	18–19	19	18
Cu(II)/phen (1:1)	0	0	0
$Cu(NO_3)_2.3H_2O$	0	0	0
cpx (5 µg/disk)	0	-	-
amp (10 µg/disk)	0	-	-

Table S9. DNA gyrase and topoisomerase IV concentrations (of *E. coli* and *S. aureus*) used in each DNA gyrase supercoiling inhibition assays and topoisomerase IV relaxation inhibition assays. Each experiment was performed using 0.5 µL of pBR322 plasmid (relaxed in the case of gyrases and supercoiled in the case of topoisomerases IV).

**Assay**	**Bacterial enzyme**	**Enzyme concentration (U)**
Gyrase supercoiling assay	*E. coli*	1
	S. aureus	1
Topoisomerase IV relaxation assay	*E. coli*	1.5
	S. aureus	2

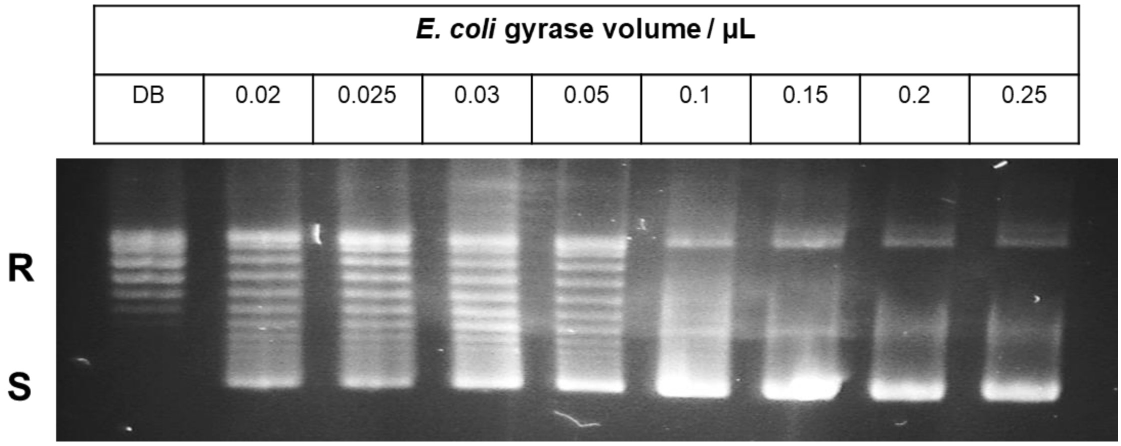

Figure S1. Activity of *E. coli* DNA gyrase in a supercoiling assay performed with 0.5 µL of relaxed pBR322 plasmid, determined in a 1% (w/v) agarose gel in TAE buffer. DB is dilution buffer, and the respective band is the negative control, containing the relaxed plasmid in the absence of the enzyme. R and S are the relaxed and supercoiled DNA bands, respectively. The results obtained for the DNA gyrases correspond to the units of the enzyme required to completely supercoil the

relaxed plasmids. The experiment was also performed with the *S. aureus* DNA gyrase supercoiling assay kit.

***E. coli* topoisomerase IV volume / µL**					
DB	0.1	0.15	0.2	0.25	0.3

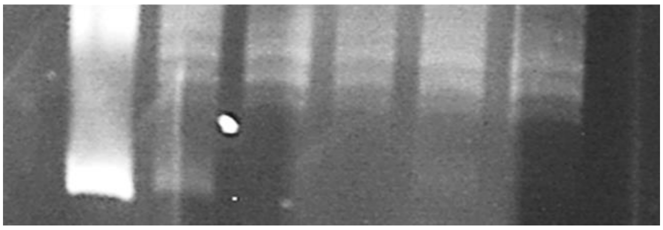

Figure S2. Activity of *E. coli* topoisomerase IV in a relaxation assay performed with 0.5 µL of supercoiled pBR322 plasmid, determined in a 1% (w/v) agarose gel in TAE buffer. DB is dilution buffer, and the respective band is the negative control, containing the supercoiled plasmid in the absence of the enzyme. The concentration of enzyme assessed for the topoisomerases reveals the amount needed to totally relax the supercoiled plasmids. The experiment was also performed with the *S. aureus* topoisomerase IV relaxation assay kit.

**DB**	**HEPES**	**cpx / uM**		**Cuspxphen / µM**					
-	+	5	50	0.5	1	5	10	50	100

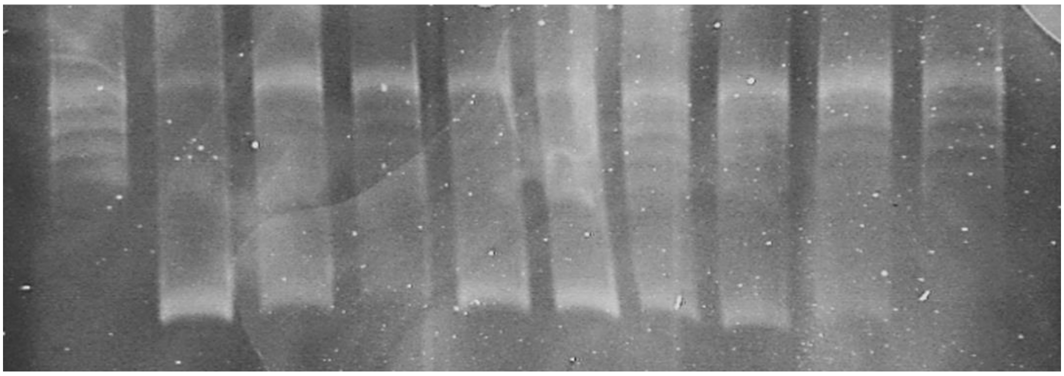

Figure S3. DNA gyrase supercoiling inhibition assay obtained for Cuspxphen as enzymatic inhibitor of the *S. aureus* DNA gyrase, performed with 0.5 µL of relaxed pBR322 plasmid, determined in a 1% (w/v) agarose gel in TAE buffer. DB is dilution buffer, and the respective band is the negative control, containing the relaxed plasmid in the absence of the enzyme. HEPES is the positive control bands containing the enzyme and the plasmid. The cpx bands represent the drug control. µM means µmol dm^{-3} and refers to the concentration of the compound. The enzymatic inhibitory activity of Cucpxphen was also evaluated.

DB	HEPES	cpx / uM		Cucpxphen / µM					
-	+	1	10	0.5	1	5	10	50	100

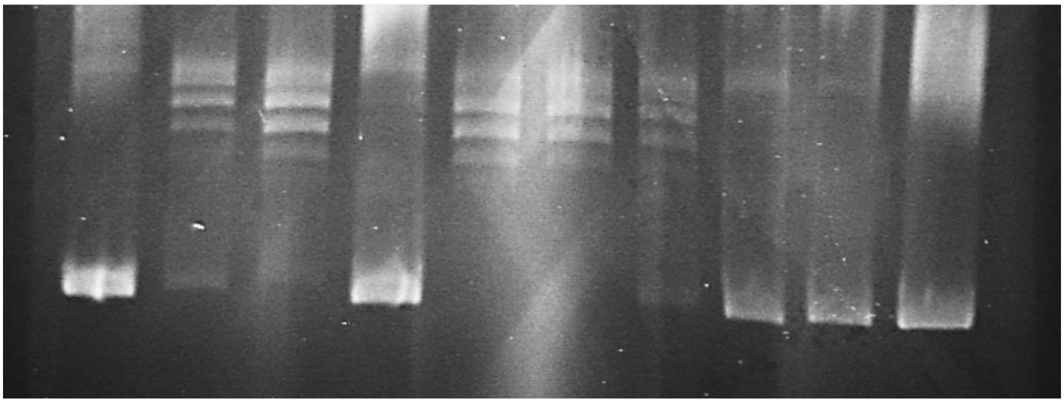

Figure S4. Topoisomerase IV relaxation inhibition assay obtained for Cucpxphen as enzymatic inhibitor of the *E. coli* topoisomerase IV, performed with 0.5 µL of supercoiled pBR322 plasmid, determined in a 1% (w/v) agarose gel in TAE buffer. DB is dilution buffer, and the respective band is the negative control, containing the relaxed plasmid in the absence of the enzyme. HEPES is the positive control containing the enzyme and the plasmid. The cpx bands represent the drug control. µM means µmol dm^{-3} and refers to the concentration of the compound. The enzymatic inhibitory activity of Cuspxphen was also evaluated.

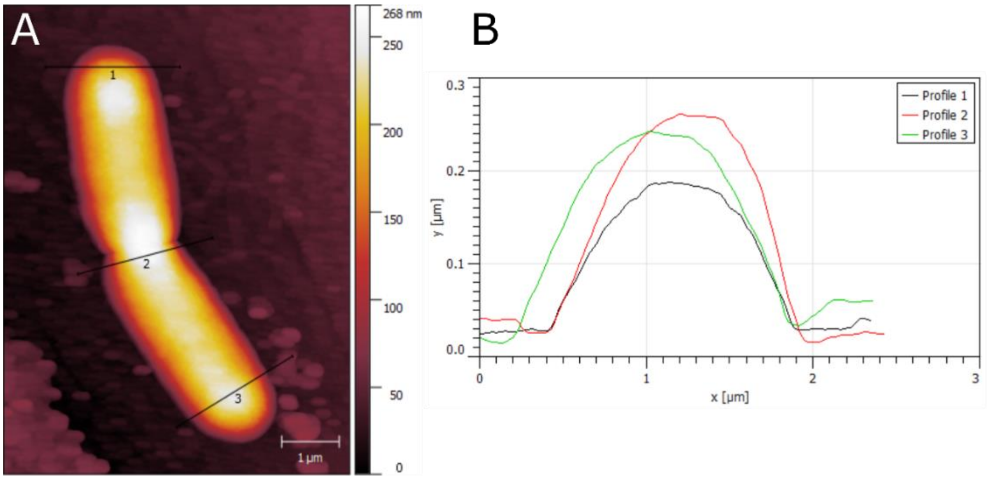

Figure S5. AFM image of *E. coli* control cells. A- height image; B- size profile of the bacterial cell measured with lines 1, 2 and 3 shown in A and generated by Gwyddion software; y axis represents cell height and x axis represents the cell size.

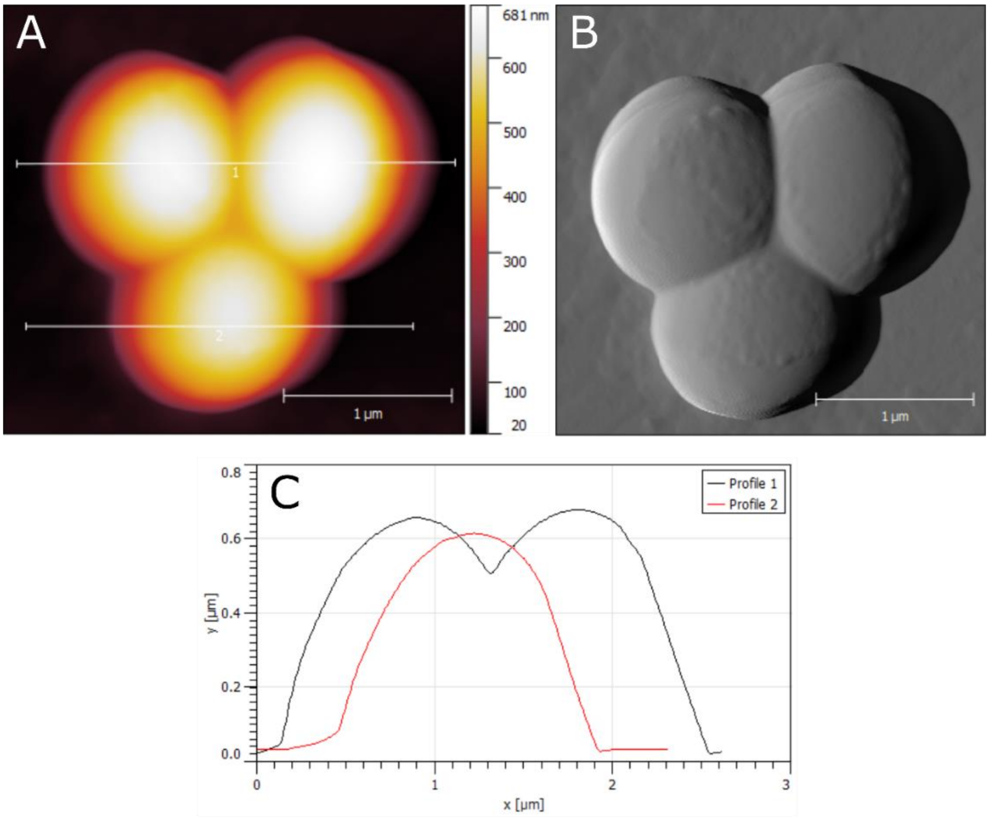

Figure S6. AFM image of *S. aureus* control cells. A- height image; B – amplitude image; C- size profiles of the bacterial cells measured at the lines 1 and 2 shown in A and generated by Gwyddion software; y axis represents cell height and x axis represents the cell size.

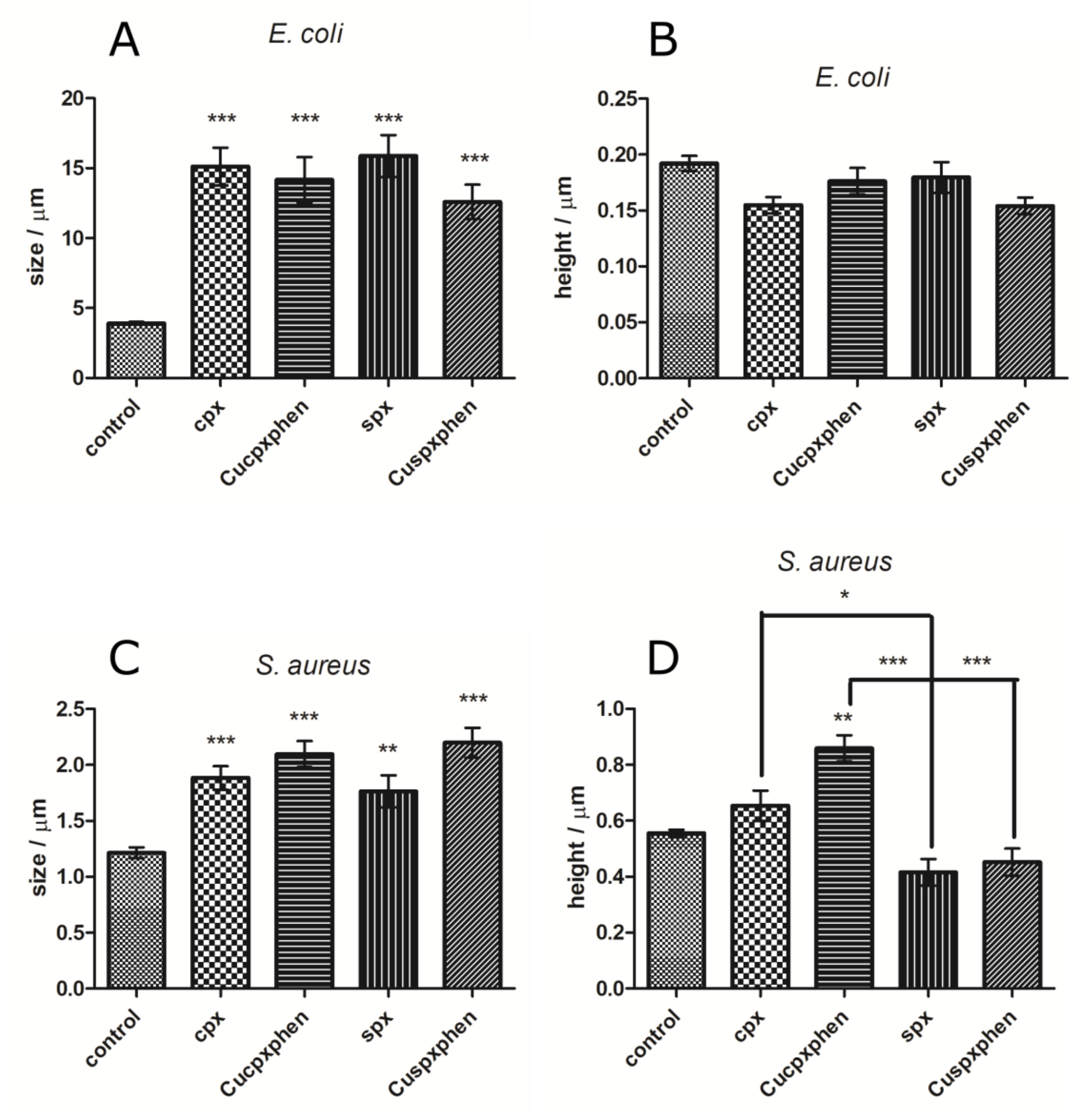

Figure S7. Sizes (Length for *E. coli* - A, diameter for *S. aureus* - C) and heights (B and D) of *E. coli* ATCC 25922 (A and B) and *S. aureus* ATCC 25923 (C and D) control and treated cells measured with AFM. The results are the average and standard error of the mean of at least 10 independent measures of individual cells from three different samples. The differences between the distributions for a $p<0.05$ were analysed using 1-way analysis of variance (ANOVA) test.

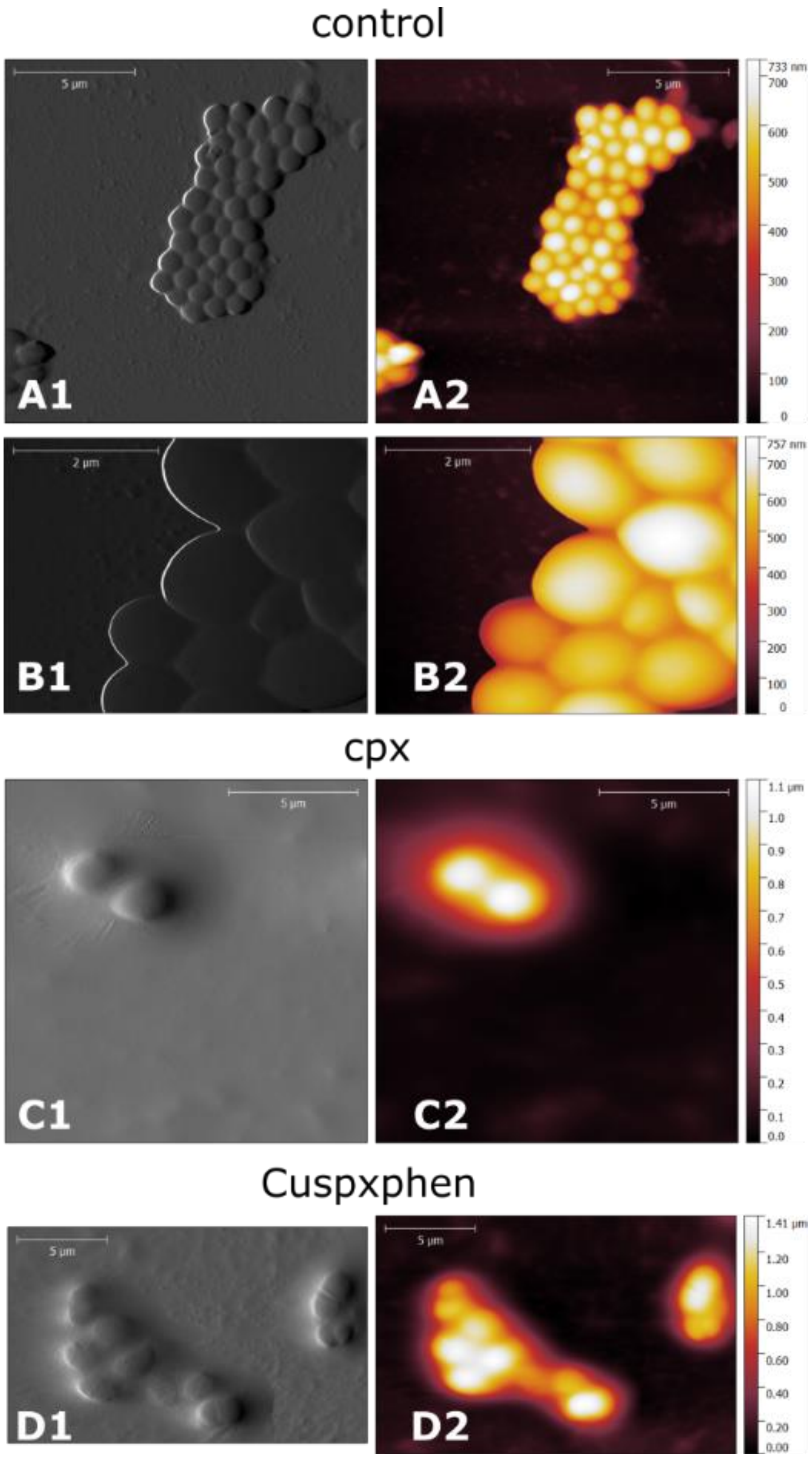

Figure S8. AFM images of *S. aureus* control cells (A and B) and cells treated with cpx (C) and Cuspxphen (D). A1, B1, C1 and D1 are phase images; A2, B2, C2 and D2 are height images. These images are representative of the multiple areas from at least three samples analysed for each condition tested.

References

1. The European Committee on Antimicrobial Susceptibility Testing (EUCAST). *Breakpoint tables for interpretation of MICs and zone diameters. Version 8.1*; http://www.eucast.org, 2018.
2. Feio, M.J.; Sousa, I.; Ferreira, M.; Cunha-Silva, L.; Saraiva, R.G.; Queirós, C.; Alexandre, J.G.; Claro, V.; Mendes, A.; Ortiz, R., et al. Fluoroquinolone–metal complexes: A route to counteract bacterial resistance? *J. Inorg. Biochem.* **2014**, *138*, 129-143, doi:10.1016/j.jinorgbio.2014.05.007.
